# The Role of Dyadic Teacher–Student Relationships for Primary School Teachers’ Well-Being

**DOI:** 10.3390/ijerph20054053

**Published:** 2023-02-24

**Authors:** Manuela Haldimann, Julia Morinaj, Tina Hascher

**Affiliations:** 1 Institute of Professional Research and Competence Development, St. Gallen University of Teacher Education, 9000 St. Gallen, Switzerland; 2 Department of Research in School and Instruction, Institute of Educational Science, University of Bern, 3012 Bern, Switzerland

**Keywords:** teacher well-being, teacher–student relationship, primary education

## Abstract

Teacher well-being is not only relevant for the effectiveness of individual teaching and student learning but also for general school quality and societal functioning, because teacher well-being is related to lower burnout risks and lower attrition. Previous research identified social relationships in school as a crucial source of teacher well-being. However, studies investigating the role of teacher–student relationships as a determining factor for teacher well-being are still scarce. This study takes a qualitative approach toward investigating the role of dyadic teacher–student relationships in teacher well-being. We analyzed twenty-six semi-structured interviews with Swiss primary school teachers, using a qualitative content analysis. The results showed that dyadic teacher–student relationships played an important to a very important role in the everyday life of teachers and were a source of both positive and negative emotions, cognitions, and physical sensations. The quality of the dyadic teacher–student relationship was reflected in the social–emotional competence of both teachers and students. Conflicts were not necessarily inhibiting teacher well-being. The findings of this study can be used to inform teacher-training institutions, as well as authorities, on how to support teachers in building relationships with their students and, in turn, foster their well-being.

## 1. Introduction

Teachers play an important role in students’ success, satisfaction, and achievement [1]. To explain intra- and inter-individual differences of teachers and their effectiveness in the classroom, teacher well-being is getting increased attention. Previous studies have shown that teacher well-being is related to more effective teaching [2,3], increased teacher self-efficacy [4], and student well-being [5]. Teacher well-being is not only relevant for teaching quality but is also interesting from a financial and economic perspective—teacher well-being correlates with a lower risk of burnout [6] and a lower intention to leave the job [7,8].

The important role of teacher well-being calls for scientific answers to the question of what contributes to its development. Among a plethora of possible well-being sources, three main sources have been identified: contextual factors, personal factors, and interactive factors [9,10]. However, neither contextual nor personal factors alone have satisfactorily explained differences in well-being between individuals, suggesting that interactional factors between the environment and a person play an important role, such as differences in subjective evaluations of situations [10]. Interestingly, while there is a large body of research on both contextual and personal factors as explanations for the development of well-being, there is a lack of research on their interaction [11]. Especially for examining psychosocial factors, such as social relationships in school, an interactive approach seems promising.

Reviews of the literature summarize the important role of social relationships as a source of teacher well-being and call for more in-depth studies [1,12,13]. An essential social contact and source of teacher well-being is the dyadic teacher–student relationship [14,15,16]. Teachers experience a large number of social interactions on a daily basis—hardly any other profession has a similar intensity of interactions. Relationship issues are often on top of the list of challenges teachers face [17,18,19]. The extent to which teacher–student relationships affect students and their school success has been widely studied and illustrates their importance for students’ academic performance [20,21,22], the development of emotional and social skills [23,24], and motivational aspects [25,26]. However, there are few studies that have examined dyadic teacher–student relationships as a source of teacher well-being [16,27,28]. Therefore, this qualitative study aims to deepen our understanding of the role of dyadic teacher–student relationships in teacher well-being from the perspective of primary school teachers. Using a qualitative content analysis, we examined interview data on the relationship between perceived dyadic teacher–student relationships and teacher well-being. A map illustrating the quality of a teacher’s dyadic relationship with his/her students in the classroom, prepared in advance by teachers, served as the basis for the interviews. Our findings contribute to advancing research on sources of teacher well-being and to the previous literature on the importance of social relationships with individual students.

### 1.1. The Dyadic Teacher–Student Relationship

The teacher–student relationship is a construct that has a variety of definitions and conceptualizations [23]. For example, the teacher–student relationship can be understood as the relationship between the teacher and the class as a whole group (e.g., global teacher–student relationship, teacher–class relationship, and collective teacher–student relationship). However, Hamre and Pianta [24] suggested describing the teacher–student relationship from a dyadic perspective that differs from the relationship between a teacher and the class on a collective level [29]. While the teacher–class relationship is frequently investigated in school and classroom climate research [30], still little is known about teachers’ subjective experiences of dyadic teacher–student relationships in the classroom [31] and especially of their significance as a source of teacher well-being. Therefore, we address dyadic teacher–student relationships that explicitly focus on the relationship between a teacher and a particular student on the individual level. Dyadic teacher–student relationships refer to cognitive schemas that both the student and the teacher develop based on previous relational experiences and that influence how relational experiences between two individuals are interpreted [16,32]. In this sense, teacher–student interactions are considered as distinct from the teacher–student relationships [16,33,34,35].

### 1.2. Teacher Well-Being

Teacher well-being has received growing attention over the past decade and has been empirically investigated using a variety of different concepts. In a recent review of the literature, Hascher and Waber [13] structured this variety of research on teacher well-being into five distinct clusters: (1) general well-being psychology, (2) positive psychology, (3) psychology of work and organization, (4) teacher well-being, and (5) health science. Despite the great diversity, most conceptualizations are based on the understanding of teacher well-being as a multidimensional construct. For the present study, we draw on research from the field of well-being psychology, which has its roots in the work of Diener [36] and his research on subjective well-being.

According to Diener [36], a person feels well when he or she experiences high levels of positive emotions and low levels of negative emotions combined with high life satisfaction. Diener introduced three characteristics that describe subjective well-being at its core. *First*, he pointed out that well-being should be regarded as subjective. Objective circumstances may influence subjective well-being, but they are not necessarily part of it. Rather, objective circumstances are evaluated differently by different people, for example, depending on their goals, values, or culture. *Second*, it must be considered that subjective well-being does not only consist of the absence of negative factors but also includes the presence of positive factors. *Third*, subjective well-being can be conceptualized differently in the temporal dimension. Drawing on research from the field of well-being psychology [37,38] and supported by Hascher’s [10,39] definition of habitual well-being in school, we define teacher well-being as a longer-term dominance of positive emotions, cognitions, and physical sensations over negative emotions, cognitions, and physical sensations in relation to the professional activity as a teacher. As suggested by Bradburn [40], positive and negative components are not understood as a continuum but rather as two independent dimensions. Furthermore, following cognitive emotion theories (e.g., [41]), a close connection between the emotional, cognitive, and physical components is assumed.

### 1.3. The Dyadic Teacher–Student Relationship as a Source of Teacher Well-Being

Several theoretical approaches and the empirical evidence suggest that the dyadic teacher–student relationship matters for the development and maintenance of teacher well-being. First, according to appraisal theories (e.g., [41,42]), the role of the dyadic teacher–student relationship in teacher well-being can be linked to the importance the teacher ascribes to a relationship. If the teacher attributes high importance to the dyadic teacher–student relationships in the classroom, it can be assumed that they are more closely linked to the well-being than if the teacher attributes low importance to them. Support for the importance of dyadic teacher–student relationships for teacher well-being can be found in theories of needs and motivation [43,44,45] and in neurobiology [46,47]. Qualitative, as well as quantitative, research findings have shown that, especially for primary school teachers, the fulfilment of the need for social belonging with students is related to teacher well-being [27,48,49]. In addition, professional identity theories propose the relevance of the dyadic teacher–student relationship for well-being [50,51,52]. Butler [50] demonstrated in a quantitative study with 530 primary and secondary school teachers that their aspiration for close and caring relationships was one of five important achievement goals.

Second, the role of the dyadic teacher–student relationship in teacher well-being may depend not only on its importance for a teacher but also on its quality. Although it can be assumed that dyadic teacher–student relationships of diverse quality are linked differently with teacher well-being, studies examining the importance of social relationships with individual students rather than their relationship with the class as a whole are still scarce. Previous empirical studies have found associations between dyadic teacher–student relationships and both positive and negative teacher emotions [33,53,54,55,56] and burnout [57,58]. Qualitative interview studies mostly elucidated opposite forms of dyadic teacher–student relationship, such as associations of teacher well-being with cognitive relationship schemas of disruptive versus non-disruptive students [55,56] or of positive and problematic dyadic teacher–student relationships [53]. However, teachers also reported dyadic teacher–student relationships that did not trigger either positive or negative emotions [56]. In studies on dyadic teacher–student relationships, as well as on the relationship with the class on a collective level, evidence of both well-being-supporting and -inhibiting relationship qualities can be found. Among the relationship qualities that support teacher well-being are students showing interest in the teacher [53], student motivation [53,59,60], respect and acceptance by students [53,61], trusting the teacher [59], closeness between students and teacher [14,33,58], honesty [61], and teachers’ caring [62]. Relationship features that inhibit teacher well-being are conflict between students and teacher [14,33,56,57,59,63], disrespect and rejection by students [53,54,59], and lack of student motivation [53,54,59]. Academic performance was shown to be less relevant in the Claessens et al. [53] study when teachers described positive and problematic dyadic teacher–student relationships. In the study by Hagenauer and Hascher [59], teachers reported joy-inducing student performance behaviors, whereas poor performance was rarely described as triggering anger. In summary, there are few studies that have examined the quality of dyadic teacher–student relationships in primary school from the perspective of teacher well-being as a multicomponent construct—including emotions, cognitions, and physical sensations.

Third, the role of the dyadic teacher–student relationship in teacher well-being may not depend solely on its importance for a teacher and its quality; rather, various teacher competences may have an impact. It might be important how teachers regulate emotions, cognitions, and physical sensations triggered by dyadic teacher–student relationships. For example, deep-acting emotion regulation strategies may help maintain a positive relationship with the student even when a situation elicits negative teacher emotions, such as anger caused by disruptive student behavior or disappointment linked to students cheating. Moreover, teachers’ social–emotional competence may be important in fostering a relationship quality with students [20,64], because relationship skills are regarded as one of the core competence areas of social–emotional competence [65]. For example, when a teacher cooperates and communicates clearly, it may help build high-quality dyadic teacher–student relationships. Moreover, teachers’ reflection on their relationship schemas [33,55,56], their ability to interrupt negative, habitualized appraisal patterns [32,66], and having relational self-efficacy [67] are suggested strategies by scholars. In dyadic teacher–student relationships, the teacher’s social–emotional competence seems to be relevant. Especially for beginning teachers, the social competence of teachers showed to be a predictor of teacher well-being that calls for increased efforts to promote social competence in university teacher education [68,69]. However, little research has been conducted on how teachers experience and deal with dyadic teacher–student relationships that inhibit or support well-being and how they build high-quality dyadic teacher–student relationships [70]. According to Hagenauer and Hascher [59], the factors to which teachers attribute successful or unsuccessful dyadic teacher–student relationships were rarely studied. They assumed that if teachers are convinced of their own ability to build relationships as a personality disposition, this could lead to an experience of fear in the case of unsuccessful dyadic teacher–student relationships. Herrmann [71] (p. 201) also pointed out that there is a high risk of professional dissatisfaction, psychosomatic stress, and even “professional incapacity” when teachers lack relationship-building skills. To summarize, these findings call for research that aims at a deeper understanding of the role of dyadic teacher–student relationships in teacher well-being from the teachers’ perspective.

### 1.4. The Present Study

This study aims at contributing to the existing literature by analyzing well-being-supporting and well-being-inhibiting dyadic teacher–student relationships from primary school teachers’ perspective. We followed a qualitative approach and aimed to extend the extant literature in two key ways. First, we considered teacher well-being as a multicomponent construct, particularly by integrating the often-neglected physical component of teacher well-being. Second, we addressed teacher–student relationships from a dyadic perspective that differs from the teacher–class relationship on the collective level. Because studies indicate differences between school levels, this paper focuses on primary school teachers. For primary school teachers, compared to secondary school teachers, dyadic teacher–student relationships have been associated with greater emotional intensity due to greater proximity [54], and social affiliation with students has been found to have greater importance for teacher well-being [49]. More precisely, in this study, we aim to address the following three research questions:

*Research Question 1:* How do primary school teachers evaluate the importance of dyadic teacher–student relationships with regard to their well-being at school?

*Research Question 2:* How do primary school teachers describe dyadic teacher–student relationships that support teacher well-being, and what professional strategies do they report for fostering those supportive dyadic teacher–student relationships?

*Research Question 3:* How do primary school teachers describe dyadic teacher–student relationships that inhibit teacher well-being, and what professional strategies do they report for coping with those inhibiting dyadic teacher–student relationships?

## 2. Materials and Methods

### 2.1. Participants

This study is part of the project “Teacher Well-Being (WoLe)”, which was carried out by the Department of Research in School and Instruction at the Institute of Educational Science at the University of Bern. We conducted semi-structured interviews with primary school teachers from the German-speaking part of Switzerland, based on a top-down sampling procedure following predefined criteria. *First*, we recruited only primary school teachers who had at least three years of teaching experience, as the literature points out that especially early career teachers explore their role as a teacher in the first years of their teaching career, which may lead, for example, to uncertainty in classroom management [72]. *Second*, all participating teachers held the position of a classroom teacher. In Switzerland, classroom teachers teach the majority of subjects, carry the main responsibility for the class, and thus seem to have particularly frequent and intensive (dyadic) teacher–student relationships [55]. *Third*, we included in the sample only teachers teaching at the upper primary level (grades 3 to 6) because their students also participated in a written survey as part of the project. The student questionnaire about school well-being and perceptions of social relationships in class required a certain level of reading skills. The results of the student survey will be presented in a separate article. In total, the purposive sample included 26 teachers (*M*_age_ = 39.8 [*SD* = 13.9]; *M*_teaching years =_ 15.87 years [*SD* = 13.4]), including 22 (84.6%) female and 4 (15.4%) male teachers. Teachers were sampled from 16 different schools from the three Swiss cantons of Berne, Lucerne, and Zurich. Teachers participated with their principal class, ranging from 14 to 24 students (*M*_size_ = 19.04 [*SD* = 2.93]). This resulted in a sample of total 495 students from 26 classes, including 244 (49.3%) female and 251 (50.7%) male students. All participating teachers stated that they felt comfortable to very comfortable at school during the weeks prior to the interview.

### 2.2. Data Collection

**Figure 1:**
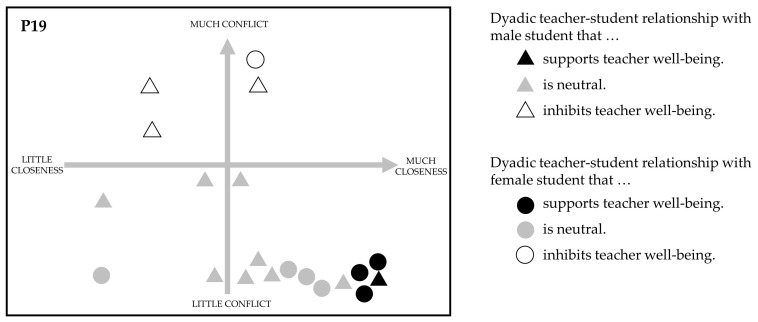
Exemplary schematic representation of the relationship map of teacher P19.

We followed a qualitative approach and conducted semi-structured interviews in German. According to Spilt and Koomen [56], interviews can elucidate the feelings, beliefs, and expectations of teachers in dyadic teacher–student relationships that cannot be captured by other measures, such as questionnaires or observations. Specifically, semi-structured interviews allow researchers an open approach to the topic while structuring the topic in terms of comparing data quality. The interviews took place before restrictions due to the COVID-19 pandemic between early January 2020 and late February 2020 and lasted about an hour. The basis for the interviews was a relationship map that teachers had individually prepared in advance (see Figure 1). With this relationship map, teachers were asked to indicate the quality of the relationship with each student in their class in a coordinate system consisting of the two orthogonal axes “much/little closeness” and “much/little conflict”. Closeness and conflict are two dimensions of the “Student–Teacher–Relationship-Scale” [73]. In a written instruction for the teachers, we included the German translation of the items [74] as a description of the two dimensions. At the beginning of the interviews, we asked teachers about their understanding of teacher well-being and how they describe their well-being in school in the past weeks. We then introduced the definition of teacher well-being as a longer-term dominance of positive emotions, cognitions, and physical sensations over negative emotions, cognitions, and physical sensations in relation to the professional activity as a teacher to foster a shared understanding of well-being throughout the interviews. Next, we asked teachers about the importance of dyadic teacher–student relationships for their well-being in school (RQ1). Then teachers identified about three dyadic teacher–student relationships on the relationship map that most supported (RQ2) or most inhibited (RQ3) their well-being. Two to three individual examples of each were then selected and discussed in depth. In addition to a general description of the selected relationship, we asked teachers to describe a typical interaction with the student and its relation to their well-being at school. By asking about typical situations, we aimed to capture regularly occurring situations and thus address manifestations in the cognitive relationship schema, as well as associations with habitual well-being. If not mentioned spontaneously, we asked teachers to elaborate on how this relationship manifests itself for the teacher (relationship schema teacher) and the student (relationship schema student), how they assess the current quality of the relationship, and what professional strategies they use in fostering or coping with those relationships.

### 2.3. Data Analysis

We analyzed and condensed the recorded and transcribed interviews in accordance with the structuring qualitative content analysis [75], using the software MAXQDA 2020. For the first research question regarding the importance of dyadic teacher–student relationships for teacher well-being, we developed no codes, and we directly analyzed and summarized the answers to the corresponding interview question. We recorded spontaneous responses indicating the level of importance (very important, important). In addition, we collected and reported exemplary positive and negative subjective well-being experiences (emotional, cognitive, and physical) across all relationship descriptions to show the variety of links between dyadic teacher–student relationships and the three qualities of well-being experience. For the second and third research questions, we developed a category system (see Table 1; for the detailed coding scheme, see Supplementary Materials Table S1) in a multistage procedure: (1) We determined the coding rules. We defined coding units as sense units, ranging from a phrase to several sentences. Due to the partly close thematic connection between the categories and the dyadic character of the dyadic teacher–student relationship (e.g., humor), we decided to allow double and multiple coding. (2) Following an iterative process, we developed the category system. First, we deductively derived categories from the literature on teacher well-being and teacher–student relationship. We created the main categories “relationship schema student” (statements of the teacher about the student) and “relationship schema teacher” (statements of the teacher about themselves), as well as the subcategory “personality”, as used in the study by Claessens and colleagues [53]. To code teachers’ personality descriptions, we used the classification of the Big Five personality traits described by Danner and colleagues [76]. In accordance with Frenzel [77] and Hagenauer and Hascher [59], we added the subcategories “relational behavior”, “motivational behavior” (including motivation), “socio-emotional behavior” (including interactions with classmates), and “performance behavior” (including performance) to the student relationship schema. We further deductively developed the subcategories of the category “relational behavior” (e.g., trust and humor) for both the student and the teacher relationship schema from extracted relationship qualities from the literature (e.g., [78]), tested them on the material, and combined them with inductively built categories. Inductively, we further differentiated the category “complementary professional strategies” of the teacher relationship schema and added the subcategories “sympathy” and “special education needs” to the student relationship schema. For each subcategory, we distinguished between positive and negative valence (e.g., respect and discipline vs. lack of respect and discipline). According to research on emotion [40,79], and specifically in the context of teacher–student-relationships [80,81], positive and negative dimensions should be considered as independent. (3) By applying and discussing the developed categories, they were constantly sharpened, and the material, if necessary, was recoded. We used the same category system for dyadic teacher–student relationships supporting teacher well-being and for dyadic teacher–student relationships inhibiting teacher well-being. By analyzing descriptions of more than one dyadic teacher–student relationship supporting or inhibiting teacher well-being per teacher, we aimed to capture the diversity of teachers’ experiences. Following Claessens and colleagues [53], the indicated frequency of the codes (see Table 1) is not based on how often a teacher used a certain code in his or her descriptions but rather on whether or not a code was present in the description of dyadic teacher–student relationships. If a teacher mentioned a certain code (positive or negative valence) one or more times, we coded it as 1. If a code was not mentioned, we coded it as 0. We explicitly point out that the frequency indication serves as an orientation and is not to be interpreted as a conclusive result. In the semi-structured interviews, not all the questions were answered in the same way.

### 2.4. Ethics and Credibility

We contacted potential participants by e-mail and invited them to take part in the study at a time convenient for them. We recruited all participants on a voluntary basis and assured them complete anonymity and confidentiality, as specified in a written consent form. Participants could withdraw from the study at any time. All teachers received the results by e-mail and were invited to participate in further discussions.

We piloted the relationship map before conducting cognitive interviews [82] with two teachers. Information regarding comprehensibility and procedure was clarified. Furthermore, we conducted the interview with the same two teachers, and we obtained indications of the comprehensibility and the time required. The interview guidelines were revised, and the interview questions were sharpened.

The first author completed most of the coding, whereby selected text passages were discussed during team meetings. Furthermore, another team member coded additionally a third of the interviews. We calculated the intercoder agreement based on the percentage of agreement (code overlap for segments of at least 90%) by using MAXQDA 2020. This resulted in a good corrected kappa value [83] of κ = 0.74.

## 3. Results

In the following sections, the main results regarding the three research questions are presented and illustrated with quotes from teachers. As the interviews were conducted in German, the selected quotes were translated into English by the first author and approved by the second and third authors. The participant number (P01–26) and the position in the transcript are indicated with examples; for instance, P03 pos. 24 indicates an exact quote from participant number 3 at position 24 in the transcript.

### 3.1. Importance of Dyadic Teacher–Student-Relationships with Regard to Their Well-Being at School—Research Question 1

All 26 teachers confirmed the importance of dyadic teacher–student relationships for teacher well-being, with 15 teachers attributing a high importance and 9 teachers attributing a very high importance to their individual well-being. One teacher explained the importance as follows:

“*When I think about the individual students, they influence my thinking. I take care of the child. Depending on the relationship, it is easier for me. I worry less. I also feel good, or happy to come to school and see the child again. I see them often, five times a week. […] Yes, the relationship is very, very important for my well-being*”

(P26, pos. 62).

In all interviews, teachers reported dyadic teacher–student relationships as a source of positive emotions, cognitions, and physical sensations. Teachers talked of *joy* when students expressed trust in them (P06, pos. 22) or provided a positive emotional response (P18, pos. 14). Teachers reported *pride* when they established a good relationship with a student (P10, pos. 37). When a student actively sought contact, *satisfaction* was reported (P13, pos. 9). Teachers mentioned *being uplifted*, as if one “had three coffees”, after a successful parent meeting (P17, pos. 24); *sleeping well* (P26, pos. 62); and feeling *relaxed* or *relieved* when students expressed gratitude after a great effort on the part of the teacher (P03, pos. 98). Certain dyadic teacher–student relationships were associated with *fun* during learning processes due to “being on the same page” (P11, pos. 114) and easily connecting to the individual child’s needs (P10, pos. 19):

“*Fun, fun, fun with learning. Right? From that point on, when the relationship is good, from that point on, you can have fun while learning. Before that, you cannot. But when you get it right, and you are both on the same page […] on the same respect level, but also on the same understanding level. From then on, you can really have that fun with learning*”

(P11, pos. 112).

Teachers also addressed dyadic teacher–student relationships as a source of negative emotions, cognitions, and physical sensations. For example, one teacher experienced it as a *burden* to maintain a time-consuming relationship with a student with special needs that left no resources to meet the needs of other students in the class (P23, pos. 117). Another teacher had the impression that a child felt uncomfortable in her presence, which led to *anxiety* (P14, pos. 88). A teacher spoke of *disgust with herself* after becoming aware that she had treated a student unfairly because of her own behavioral patterns and triggers (P11, pos. 84). A teacher reported *frustration* when no progress had been made in the quality of the relationship despite many efforts (P10, pos. 74). A teacher talked about one dyadic teacher–student relationship that led to a *feeling of powerlessness* on the part of the teacher as to how best to support this student (P16, pos. 213). A difficult dyadic teacher–student relationship led to teachers’ *worrying* in the evening about the causes of existing conflicts (P21, pos. 94) or about how to help a child in a difficult situation (P15, pos. 17). The extent to which a difficult dyadic teacher–student relationship can affect teacher well-being was illustrated by the description of a teacher who has taught a student with difficult socio-emotional behavior and who strongly criticized the teacher. The teacher described becoming *ill*, suffering from various *colds,* and *not wanting to get up anymore* (P14, pos. 158). Some teachers also reported that a difficult dyadic teacher–student relationship had almost caused them to leave the teaching profession in the past, as expressed by one young teacher as follows:

“*If I regularly noticed that I was not getting along with the children, then that would be a reason for me not to do this job*”

(P02, pos. 100).

The reported positive and negative effects of dyadic teacher–student relationships on teacher well-being should not be understood as an exhaustive list; rather, they represent the multifaceted spectrum of dyadic teacher–student relationships as a source of teacher well-being.

### 3.2. Dyadic Teacher–Student Relationships That Support Teacher Well-Being—Research Question 2

We describe teacher well-being-supporting dyadic teacher–student relationships based on (1) how teachers located those relationships on the relationship map and (2) the representation of the three most prevalent subcategories of each category of the student and teacher relationship schemas. Table 1 shows how frequently teachers mentioned the subcategories in their talk about dyadic teacher–student relationships that support teacher well-being. It has to be noted that the descriptions of the relationship schema of the student and the teacher are closely linked due to the interactional character of dyadic relationships, and thus their separation should be considered to be a heuristic.

#### 3.2.1. Characterization on the Relationship Map

When asked to identify around three dyadic teacher–student relationships that support teacher well-being the most, teachers classified a total of 107 relationships, with 45 (42.1%) being a relationship with a male student and 62 (57.9%) being a relationship with a female student (see Table 2). Of those dyadic teacher–student relationships, 15 were located by teachers in Quadrant I and therefore characterized as relationships with much closeness and much conflict. None of the relationships was in Quadrant II, with little closeness and much conflict. While 5 of the relationships were in Quadrant III, with little closeness and little conflict, 87 of the relationships were in Quadrant IV, with much closeness and little conflict.

#### 3.2.2. Relationship Schema Student 

When teachers talked about dyadic teacher–student relationships that support their well-being, most teachers talked in terms of students’ *personality, intelligence, and special education needs* in regard to extroverted students (*n* = 11), students high in agreeableness (*n* = 7), and students with high intelligence (*n* = 8). For example, one teacher spoke of working with an intelligent student leading to satisfaction and joy:

“*He is just clever. We can discuss or explain things at a slightly higher level than with other kids. It’s just exciting to not only talk about the 4th grade material*”

(P21, pos. 122).

Key discussion topics related to student *relationship behaviors* included students’ active relationship building with the teacher (*n* = 17), shared humor (*n* = 10), and issues related to lack of respect for the teacher and disciplinary problems (*n* = 11). Students’ active relationship building with the teacher was experienced by one teacher as follows:

“*I come into the classroom and he is already running toward me with something that he either wants to show me what he has done or that he wants to ask me (laughs)*”

(P14, pos. 108).

Regarding the *motivational behavior* of the students, teachers (*n* = 10) talked about motivated students who like coming to school, are concentrated on their work, contribute their own ideas to the lessons, or try to implement tips from the teacher. Few teachers (*n* = 3) spoke of unmotivated students. Motivated students can come along with a good feeling for the teacher:

“*I can rely on her, she is doing her best. And she also comes up with questions when she does not understand something. And that is a very good feeling for me. So I do not have to ask at home: ‘Oh, why did she only do three tasks and they are all wrong?*’”

(P04, pos. 39).

Concerning the *socio-emotional behavior* of the students, positive interactions with classmates (*n* = 7) were described as having friends in class and being integrated. On the other hand, some teachers (*n* = 4) talked about students involved in negative interactions with classmates. For example, for positive interactions, one teacher explained that a student was an important tie in the social network of the class, contributing to its cohesion and therefore supporting her well-being:

“*That’s what this class needs, maybe that’s what makes me feel good, because she’s so important, such a rock for the class. She holds the class together a bit, which is important*”

(P20, pos. 41).

Regarding *performance behavior*, the high performance of students (*n* = 11) and few students showing low performance (*n* = 3) were mentioned. High performance led to joy and pride among teachers, as the following example illustrates:

“*I cannot take credit for it, but I am proud of this girl. I can also praise her a lot and that is what we teachers extremely like to do*”

(P20, pos. 43).

#### 3.2.3. Relationship Schema Teacher

Teachers rarely talked about their own *personality* in the context of well-being-supporting dyadic teacher–student relationships. Teachers described themselves in terms of extraversion (*n* = 1), agreeableness (*n* = 1), and as being emotionally stable or calm (*n* = 1).

“*Then I think maybe it has a little bit to do with my nature. Well, I am a very calm person and I am not someone who panics easily*”

(P24, pos. 66).

When describing their *relationship behaviors*, teachers most often talked about their active relationship building with students (*n* = 15). Extracurricular activities, such as school trips, provided opportunities for initiating personal conversations with students. However, even in school, “chitchat times” before or after class can also be a platform for bonding. One teacher shared how she actively connected with the students when greeting them in the morning:

“*I just ask: ‘How are you?’ in the morning. Or: ‘What did you eat?’ Or: ‘How is your hamster?’ Or yes, often it is conversations like that in the morning when they come. I think that is where I can have a lot of influence*”

(P15, pos. 89).

Furthermore, the understanding of students’ behavior (*n* = 14) was an important topic. Through the understanding of a student’s response pattern, anger can be prevented. One teacher shared that a first step in gaining understanding for her begins with greetings in the morning. The look in the eyes, i.e., blurred or clear; and the posture, i.e., relaxed or sluggish, provided the teacher with important clues about the students’ well-being. However, to develop an understanding of students, teachers pointed out the importance of looking at their own behavior patterns, as illustrated in the following quote:

“*And I think to really sit down and deal with these children who evoke something in you that actually has to do with you and your history. […] how can I change the focus and meet the child in a new way*”

(P11, pos. 82).

Almost with the same frequency as understanding the student’s behavior, teachers talked about their (professional) support of the student (*n* = 13). When a student needed a lot of support, the relationship could be strengthened through the intensive coaching, leaving room for progress and a sense of influence and satisfaction for the teacher. Even if a teacher had not yet found the right support measures but supporting a student was perceived as challenging and not overwhelming, teachers reported well-being in the long run when they eventually succeeded:

“*Challenge, self-reflection, frustration, but it is exactly this frustration that makes me reflect […] It is on so many levels afterwards where you feel well-being, that you have […] you have chosen the right path*”

(P13, pos. 75).

Concerning *complementary professional strategies*, teachers most frequently spoke of successful parental involvement (*n* = 13). Clarifying parent meetings, which represented a turning point in the dyadic teacher–student relationship, as well as generally cooperative and supportive parents, were mentioned. Teachers described that, on one hand, the quality of parental involvement may influence teachers’ perceptions of the relationship when perceiving parents’ appreciation. On the other hand, teachers reported that they think the quality of parental involvement also influences students, as they came to school less skeptical or were able to open up to the teacher:

“*With this child it was important that I had a good relationship with the mother first. I have the feeling that as soon as he knew ‘Mommy says at home, it’s ok at school, and the teacher does it well’, then he was able to open up*”

(P23, pos. 101).

Other strategies mentioned by teachers included giving the relationship enough time to develop or improve (*n* = 6) and collaborating with (special education) teachers and specialists (*n* = 3) who supported their understanding of student’s behavior based on additional information.

### 3.3. Dyadic Teacher–Student Relationships That Inhibit Teacher Well-Being—Research Question 3

We describe teacher well-being-inhibiting dyadic teacher–student relationships based on (1) how teachers located those relationships on the relationship map and (2) the representation of the three most prevalent subcategories of each category of the student and teacher relationship schemas. Table 1 shows how frequently teachers mentioned the subcategories in their talk about dyadic teacher–student relationships that inhibit teacher well-being. The results are listed in Table 1, in the column “Type of relationship—Inhibiting”, on the left side of Table 1 for the relationship schema student and on the right side of Table 1 for the relationship schema teacher. For each relationship quality of the subcategories, we distinguished between teachers talking in positive (+) or negative (-) valence (e.g., respect and discipline vs. lack of respect and discipline). It has to be noted that the descriptions of the relationship schema of the student and the teacher are closely linked due to the interactional character of dyadic relationships, and their separation should therefore be considered to be a heuristic.

#### 3.3.1. Characterization on the Relationship Map

When asked to name two to three dyadic teacher–student relationships that inhibit teacher well-being the most, teachers classified a total of 74 relationships, with 53 (71.6%) being a relationship with a male student and 21 (28.4%) being a relationship with a female student (Table 3). Of those dyadic teacher–student relationships, 25 were located by the teachers in Quadrant I and therefore were characterized as relationships with much closeness and much conflict. A total of 22 of the relationships were located in Quadrant II, with little closeness and much conflict. In addition, 17 of the relationships were in Quadrant III, with little closeness and little conflict, while 5 of the relationships were in Quadrant IV, with much closeness and little conflict.

#### 3.3.2. Relationship Schema Student

When teachers talked about dyadic teacher–student relationships that inhibit their well-being, most teachers talked in terms of students’ *personality, intelligence, and special education needs* in regard to emotionally unstable students (*n* = 7), introverted students (*n* = 4), and students with suspected or confirmed *special education needs* (*n* = 7), such as attention deficit hyperactivity disorder. One teacher expressed disappointment with an introverted student who solved tasks like a “machine”:

“*It’s simple—there’s just no output coming, no emotion from him, just really very little. Maybe I am a little disappointed in the sense of ‘Hey, you could do so many cool things to talk about, also with the other kids, but you ONLY sit there and concentrate on yourself’. Maybe it’s selfish of me too, but I just think that would be so nice*”

(P21, pos. 160).

Major discussion topics related to the *relationship behavior* of the students included lack of students’ respect for the teacher and discipline (*n* = 18), as well as active (*n* = 11) and non-active (*n* = 11) relationship building with the teacher. One teacher worried about how her own feelings were affected by a student’s provocative behavior, causing her to almost explode.

“*What worries me is that it can trigger feelings in me so that I really almost explode. And how she can influence me with her behavior […] That makes her really challenging. Sometimes I am standing here and she is doing something at her desk two steps away from me. Things like eating at school, or playing with her toys, even though we agreed that they are not allowed. She does that often in a row and really provokes me*”

(P19, pos. 105).

Regarding the *motivational behavior* of the students, teachers (*n* = 11) talked about unmotivated students who forgot to do their homework, could not find it, or did not make an effort to solve the tasks set by the teacher. This led to anger, annoyance, and frustration on the part of the teacher, because available resources were not being used, and this usually meant extra work for the teacher, as illustrated in the quote below. Nevertheless, teachers (*n* = 9) also talked about motivated students.

“*Sometimes I get angry at the child because I think we have already discussed doing a reading comprehension once a week so many times. I asked, ‘And, did you do it now?’ and he answered ‘No, I do not feel like it!*’”

(P25, pos. 22).

Concerning the *socio-emotional behavior* of the students, more teachers described students involved in negative interactions with classmates (*n* = 9) inside and outside the classroom (e.g., on the way to school) than students involved in positive interactions (*n* = 2). Although aggression was not directly expressed at the teacher, it had a negative impact on the dyadic teacher–student relationship and could lead to sleepless nights, as illustrated by the following example of an incident outside of the classroom:

“*One incident that shocked me was that he was fighting with a boy so much that he just lashed out. Then the grandmother of the other boy came […] This boy threw the worst things at her like ‘Your son deserves to die!’ […]. I did NOT know what to do anymore. And when you get into a situation like that, I realized that my relationship with him changed too. It’s not his fault, it was a change. It was not against me, but in some ways, it was against me. It was extremely challenging, I had sleepless nights*”

(P20, pos. 49).

Regarding *performance behavior*, low performance of students (*n* = 10) but also good performance (*n* = 7) was described. Low performance led to nervousness in one teacher:

“*But somehow I have the feeling that the child cannot mobilize its full resources. And that makes me a little nervous*”

(P20, pos. 43).

#### 3.3.3. Relationship Schema Teacher

Teachers did not talk (*n* = 0) about their own *personality* in the context of well-being-inhibiting dyadic teacher–student relationships. When describing their *relationship behaviors*, teachers most often expressed an understanding of student behaviors (*n* = 22). Teachers talked about the fact that it is hardly possible to have an equally good relationship with all students, that conflicts are part of daily school life, and that not all students have a need to be close to the teacher, things they first had to learn to accept. Meeting students’ behavior with understanding was a challenging process for some teachers and required daily effort. Teachers most often explained difficult student behavior in terms of socialization in the parental home. For one teacher, getting to know parents led to increased understanding of a student’s behavior and helped her find her own “peace”:

“*Much of what the parents represent you then recognize in the child, including the problems that are passed on or lived through the child. And much can then be explained in the child. Sometimes it helps to find peace and say, ‘Okay, I just need to show the child that there is something else’. And suddenly the child can develop. You know where the problem lies*”

(P11, pos. 200).

Furthermore, teachers claimed that not being able to adequately support a student (*n* = 18) resulted in anger at themselves and feelings of helplessness. In the case of lack of progress, teachers talked about resignation at a certain point, potentially leading to leaving a student on the side. Teachers also reported that when a student needed a high level of support, they were unable to adequately support other students in the class. This generated discomfort and a feeling of being drained, as illustrated in the following quote:

“*He is a totally interesting boy and I like him, really a lot. That is the dilemma. He is not well supported; he needs so much support that we cannot give him because he does not have the right for extra hours. We will probably get a class assistance because he is pretty much draining us with the attention he needs. […] That is where I do not feel comfortable anymore because I realize I am not doing justice to the other students, because I am spending so much time on him. This is difficult for him and also for the other children*”

(P20, pos. 49).

Disciplinary interventions were the third main topic (*n* = 14), whereby teachers described different methods ranging from small interventions (e.g., a look) to severe interventions (e.g., a time-out in the principal’s office). Those measures were accompanied by a range of sensations such as pity or rage:

“*Anger. I get really ‘Ahh.’ […] I then explode. I really explode by saying ‘It just does not work like that’. But then it is over. And then I am fine again*”

(P04, pos. 45).

Concerning *complementary professional strategies*, teachers most frequently talked about collaboration with (special education) teachers and specialists (*n* = 8). It was perceived as helpful to be able to exchange ideas about difficult situations, to be able to delegate something from time to time, or to get valuable tips. In addition, teachers (*n* = 6) mentioned difficult parental involvement, for example, because parents did not support the disciplinary interventions of the teacher. One teacher described her difficulties in not letting her relationship with the student be negatively affected by her poor relationship with parents:

“*Lately I had a case where I really had to turn quite strongly away from the mother because I realized that her dislike of me was influencing my relationship with the student. I really had to take a pair of scissors in my hand, purely mentally, cut the umbilical cord and say: ’This is a person here, this has nothing to do with my student over there.’ And that was hard. That was hardcore, honestly. I have never experienced that so strongly before, I was a bit scared*”

(P11, pos. 200).

In addition, resetting the relationship and giving it another chance (*n* = 5) was also mentioned as a strategy to deal with well-being-inhibiting dyadic teacher–student relationships:

“*A principle of mine is ‘Never start the day on yesterday’s broken glass’. Every day is a new chance for every child. This is an important principle, and I have had good experiences with it. We can have conflicts, but the children also have to learn that they are over at one point and then it is good again*”

(P12, pos. 63).

## 4. Discussion

This study aimed to understand the role of dyadic teacher–student relationships in teachers’ well-being. We used a qualitative approach to examine how Swiss primary school teachers describe the importance of dyadic teacher–student relationships for their well-being at school (RQ 1). We also investigated how they describe dyadic teacher–student relationships that support or inhibit teacher well-being and what professional strategies they report to foster or cope with those dyadic teacher–student relationships (RQ2 and RQ3). We conceptualized teacher well-being as a multicomponent construct that includes emotions, cognitions, and physical sensations and focused specifically on teacher–student relationships from a dyadic perspective, i.e., between the teacher and an individual student. Valuable implications for research and teacher education institutions, as well as authorities supporting teacher well-being, can be drawn from the findings.

Regarding the first research question, the results confirmed that dyadic teacher–student relationships play an important role for teachers in everyday school life and are a source of a variety of positive and negative emotions, cognitions, and physical sensations. This finding aligns with the results from previous studies examining the role of the fulfilment of the need for social belonging in teacher well-being [48,49]. Interestingly, teachers mentioned fewer associations with physical sensations in comparison to emotions and cognitions. One possible explanation for this finding might be that teachers experienced, in general, a high level of physical strain in everyday school life and therefore considered physical sensations in the context of social relationships to be normal and part of the teaching profession. Another explanation is that it might be more difficult for teachers to be aware of possible associations between their physical sensations and dyadic teacher–student relationships. Probably, neurobiological responses in the context of social relationships [46,47] tend to escape teachers’ awareness.

Regarding the second and third research questions, examples of dyadic teacher–student relationships supporting or inhibiting teacher well-being varied across individuals. Interestingly, both positive and negative relationship qualities (e.g., conflict and lack of motivation) were present in both supporting and inhibiting dyadic teacher–student relationships. We therefore suggest that the combination of different relationship qualities is crucial. According to the multicomponent construct of well-being, we hypothesize that a dyadic teacher–student relationship supports teacher well-being when teacher well-being-supporting qualities outweigh the inhibiting ones. As suggested by other authors [80,81], those two dimensions should be considered independent of each other. Studies that capture the relationship quality by using only one dimension may underestimate the complexity of social relationships. However, the role of the intensity with which different relationship qualities impacted teacher well-being could not be investigated based on the available data. Theoretically, it could be argued that well-being-inhibiting relationship qualities could have a stronger effect than well-being-supporting relationship qualities [18,84]. There is also a need to clarify to what extent teacher well-being-supporting relationships can be distinguished from “successful” or “strong” relationships.

Despite the diversity in teachers’ description of dyadic teacher–student relationships, some differences between relationships that support and relationships that inhibit teacher well-being were discovered. Almost all of the dyadic teacher–student relationships described as supporting teacher well-being were characterized by intense closeness. Characteristics of those relationships included students’ active relationship building with the teacher, motivation, positive interactions with classmates, and humor, indicating the important role of students’ active involvement in interactions with teachers. Similar student behavior emerged in Claessens et al.’s [53] study of “positive” relationships. Interestingly, nearly half of the teachers also described issues related to lack of respect for the teacher and disciplinary problems. This raises the question of why teachers characterized these relationships as supporting their well-being at school? First, the combination of conflict and closeness supports results of Newberry and Davis [63] that, compared to disruptive behavior, student pressure for a close relationship with the teacher has an important role in the emergence of emotionally positive teacher–student relationships. Second, students’ relational behavior in conflicts (e.g., honesty, clear communication of their needs, and trust in the teacher) and teachers’ professional strategies for relationship building likely played a role. Possibly, students who express their needs and actively interact with teachers contribute to the well-being-supportive relationships, even when this interaction is sometimes conflictual. In terms of professional strategies for fostering teacher well-being-supportive relationships, teachers mentioned (1) being appreciative and understanding toward students, (2) establishing good collaborations with special education teachers and involving parents, and (3) using time slots outside of regular school hours (e.g., extracurricular activities) for relationship building. Some teachers also reported that dyadic teacher–student relationships, perceived as a negative source of teacher well-being, prompted their individual professional learning and therefore had a longer-term positive effect on their well-being as a teacher. Having such a growth mindset seems specifically interesting from the perspective of continuous professionalization. As in Hargreaves’ [54] study, teachers experienced pride and satisfaction from building good dyadic teacher–student relationships, which, in the sense of Lortie [85], can be viewed as “psychic rewards” of their daily work. Isenbarger and Zembylas [86] also observed in a longitudinal case study that personal satisfaction was experienced when achieving progress within difficult situations with students.

Looking at the relationship maps, the first thing that stands out is that there were three times as many well-being-inhibiting dyadic teacher–student relationships with male students than with female students. Similar findings have been found in other studies, in which teachers more often referred to relationships with male students when selecting high-conflict relationships (e.g., [33,55]). Because the key topic in the description of teacher well-being-inhibiting dyadic teacher–student relationships was conflict, several explanations are possible. First, regarding the personality and relationship behavior of the student—in comparison to dyadic teacher–student relationships that promote well-being—teachers more often reported a lack of emotional stability and honesty. Moreover, poor communication of needs by students was reported, which would be an important relationship skill [65]. Second, a lack of student motivation was present in almost half of teachers’ descriptions. This triggered anger and frustration, which is consistent with existing research findings [53,54,59]. Third, nearly one-third of teachers described negative interactions with classmates. Even though the teacher was not directly involved, the teachers’ goal of creating a good classroom climate was threatened [77]. In sum, teachers may ascribe those behaviors more to male than female students, and this, in turn, leads to more well-being-inhibiting dyadic relationships with male students.

Interestingly, conflict and closeness were not mutually exclusive. This can be interpreted as an indicator of high social–emotional competence of teachers. Almost all teachers expressed understanding for a student’s situation and described how they used cognitive reappraisal (e.g., [87]) and regulated their emotions (e.g., [88]). On the other hand, two-thirds of the teachers expressed a feeling of providing inadequate support to the students. It is therefore not surprising that collaboration with (special education) teachers and specialists was mentioned as a professional strategy. According to the Collaborative for Academic, Social, and Emotional Learning (CASEL) [65], asking for help when needed is an important relationship skill. Professional collaboration contributes to an understanding of difficult student behavior, allowing for closeness despite conflict.

While the teachers, despite the difficulties, described relationships with positive development or at least had hope for it, some teachers also reported rigid negative interaction patterns when the same conflicts occurred again and again or (professional) support did not bring progress. Trapped in such patterns of relationships, a certain resignation became apparent among teachers. As reported by Newberry and Davis [63], teachers might distance themselves from a student or minimize interactions because of their own vulnerability. “Never start the day on yesterday’s broken glass”—in times of lack of progress and setbacks, teachers reported the important professional strategy of starting each day anew. When teacher’s relational aspirations were repeatedly not met, the relationships remained fraught with negative emotions, cognitions, and physical sensations. This might be particularly critical for teachers ascribing to well-functioning dyadic teacher-student relationships a high importance for student’s school success.

Despite experiencing well-being-inhibiting dyadic teacher–student relationships, all teachers reported feeling well or very well at school. Dyadic teacher–student relationships should be viewed as only one source that, in combination with other aspects, influences teacher well-being [1,13]. Therefore, promoting well-being should not be restricted to a teacher’s individual responsibility. Rather, measures should be taken holistically at all school levels [1]. Sharing understanding and sources of well-being [89] can be a first step toward co-constructively designing a school that aims to promote the well-being of all stakeholders.

### 4.1. Implications for Practice

Our findings demonstrate the important role that dyadic teacher–student relationships play in teacher well-being. As our results show, dyadic teacher–student relationships can be a source of positive and negative emotions, cognitions, and physical sensations, and, therefore, they can be a resource for or a threat to teachers’ well-being. It is therefore essential that relationship building is not left to chance and professional strategies for developing and dealing with dyadic teacher–student relationships are addressed as early as possible, as part of the initial teacher education. In particular, (pre-service) teachers should be encouraged to train their social–emotional competence (e.g., [68]). For example, participating teachers reported that creating and reflecting on the relationship map was an enriching and valuable experience. After entering the profession, the relationship map might therefore be a suitable tool for teachers to reflect on their relationships with their students and a good starting point to work on habitualized unproductive attribution patterns [42]. Interventions that have already been tested for effectiveness, such as the “Relationship-Focused Reflection Program” [90], or more universal approaches implemented to all students at the school or class level (for a meta-analysis see [91]) are other options that can be implemented by teacher education institutions or schools. Because we understand teacher–student relationships as dyadic, not only the social–emotional competence of teachers but also the promotion of the social–emotional competence of students should be prioritized. Our findings also revealed that professional collaboration was relevant for establishing and managing dyadic teacher–student relationships. High levels of teachers’ social–emotional competence might be beneficial for building valuable collaboration with (special education) teachers, specialists, and parents. This exchange can offer teachers the opportunity to reflect with an external person on their own relationship patterns and to develop alternative ways of acting. Required resources and structures are needed to establish those partnerships. Furthermore, teachers indicated their inability to adequately support students when their academic performance was very high or very low. Strengthening subject didactic competencies could improve teaching quality, which could lead to greater teacher well-being. Teachers also described that, in their first years of professional activity, resources were mostly absorbed by preparing and teaching different topics. It therefore seems important that on-the-job training is also offered for experienced teachers. As they gain confidence and establish a routine in teaching, they have more resources available to engage in social relationships with students and implement new impulses. Developing a growth mindset and considering challenging relationships as a valuable opportunity for their own professionalization might be beneficial. Moreover, our study gives insight into the complexity and daily effort of relationship building for teachers (e.g., self-reflection and work collaborations). When considering the idea of teachers establishing and maintaining dyadic teacher–student relationships with all students in class, it seems beneficial to provide stable class compositions [20,64].

### 4.2. Limitations and Future Research

This study has several limitations. *First,* individual understanding of different relationship qualities may vary among teachers [63]. In the case of the relationship map, we addressed this issue by integrating the German items of the Student–Teacher Relationship Scale [73] by Milatz and colleagues [74] to describe the dimensions “conflict” and “closeness” in the written instructions. *Second,* the results aimed at capturing the cognitive representations of the dyadic teacher–student relationship from teachers’ perspective, which must be distinguished from the actual interactions, as well as the students’ perspective. Previous studies revealed differences between teachers’ and students’ perception of their relationship (e.g., [92,93]). In addition, it would be insightful to examine the extent to which dyadic teacher–student relationships supporting teacher well-being are also beneficial for students or whether there might be dissimilarities. Future research should include students’ perceptions and, ideally, a third, independent observer perspective. This would allow us to examine the possible discrepancies between external and self-perception [80] and possible causes, as well as consequences for teacher well-being. There are clues in our material that a divergence can greatly unsettle teachers. *Third,* as it is common in qualitative research, the results are based on a small and non-representative sample. Therefore, the results should be interpreted as the subjective experiences of twenty-six primary school teachers in their specific environment. All teachers assessed their well-being in school as high to very high, and there were indications of high social–emotional competence. *Fourth*, due to the face-to-face interviews, it might be that some statements were influenced by social desirability. We attempted to prevent this by maintaining an open and comfortable atmosphere during the interviews. *Fifth,* long-term studies are needed to clarify possible causalities in the role of dyadic teacher–student relationships in teacher well-being.

With this study, we hope to make a small contribution, and further research is needed. We focused on the role of dyadic teacher–student relationships in teacher well-being. The role of teacher–class relationship on a collective level needs to be clarified. Moreover, there is a need to theoretically, as well as empirically, explore the relationship between those two types of relationships and how they differ from concepts such as classroom management or classroom climate. In addition, the interview data indicated interindividual differences in teachers’ descriptions of well-being-supporting and -inhibiting dyadic teacher–student relationships (e.g., preference for close, conflict-rich or distant, and conflict-free dyadic teacher–student relationships). Teachers sometimes reported in the interviews that they experienced relationships differently than other teachers teaching the same class (e.g., subject teachers). This has also been observed in other studies (e.g., [55,94]). A potential focus for investigating those differences might be possible associations with the teacher’s professional experience. Studies showed that perceptions of conflict and closeness varied as a function of professional experience [92] and descriptions of positive dyadic teacher–student relationships differed [53]. Studies also showed that, especially for teachers with less professional experience, social affiliation with students was more strongly associated with teacher well-being [48,49]. Following the study of Isenbarger and Zembylas [86], a longitudinal perspective would help to understand intraindividual differences and changes of the relationships over time (e.g., based on the relationship map) in relation to teacher well-being. Case studies would also allow for a more in-depth investigation of the combinations and interplay of individual relationship qualities within relationships. More research is also needed based on a sample including teachers with low well-being.

## 5. Conclusions

The present study illustrates the role of dyadic teacher–student relationships in teacher well-being. It gives insights into the variety of emotions, cognitions, and physical sensations triggered by the relationships and the importance teachers ascribed to them. Descriptions of teacher well-being-supporting and -inhibiting dyadic teacher–student relationships were found to be complex, with certain relationship qualities (e.g., conflict and lack of motivation) being present in both types of relationships. This supports the idea of treating relationship qualities of positive and negative valence as independent. We discovered some key differences in both types of relationships (e.g., conflict and closeness). To establish and maintain teacher well-being in the context of dyadic teacher–student relationships, teachers’ social–emotional competence and professional strategies, such as successful parental involvement and collaboration with (special education) teachers and specialists, seem impactful. It would be beneficial if teacher education institutions and authorities actively supported teachers in building high-quality relationships with their students by providing learning opportunities, time, and resources. Support in this area would be beneficial not only to teachers’ well-being and, thus, their retention in the teaching profession but also to students’ academic success.

## Figures and Tables

**Table 1:** Coding scheme and results of frequency analysis.

*Relationship* *Schema Student*	Type of Relationship				*Relationship* *Schema Teacher*	Type of Relationship			
	Supporting		Inhibiting			Supporting		Inhibiting	
*Personality, intelligence, and special education needs*	+	−	+	−	*Personality*	+	−	+	−
Emotional stability	3	5	7		Emotional stability	1			
Extraversion	11	2		4	Extraversion		1		
Agreeableness	7		2	1	Agreeableness	1			
Conscientiousness	5		2		Conscientiousness				
Openness	5				Openness				
Sympathy	5		2	1					
Intelligence	8		2						
Special education needs	1		7						
*Relational behavior*	+	−	+	−	*Relational behavior*	+	−	+	−
Respect and discipline	9	11	2	18	Honesty	6		1	4
Honesty	8			6	Understanding	14		22	9
Clear communication of one’s own needs	6	1	2	9	Appreciation	11		6	
Trust	8		1	1	Trust	3		1	3
Willingness to help	9			1	Humor	10			
Gratitude	4		1		Reliability	3			
Humor	10			1	Justice	3		2	
Active relationship building with teacher	17	2	11	11	Active relationship building with student	15	2	11	11
					Disciplinary interventions	7	4	14	
					Positive reinforcement	4		7	
					(Professional) support	13		2	18
*Motivational behavior*	+	−	+	−	*Complementary professional strategies*	+	−	+	−
Motivation	10	3	9	11	Parental involvement	13		4	6
*Socio-emotional behavior*	+	−	+	−	Collaboration with (special education) teachers and specialists	3		8	
Interactions with classmates	7	4	2	9					
*Performance behavior*	+	−	+	−	Time to develop or improve the relationship	6		4	
Performance	11	3	7	10					
					Reset of the relationship			5	

Note: + = positive valence (e.g., respect and discipline); − = negative valence (e.g., lack of respect and discipline).

**Table 2:** Locations of dyadic teacher–student relationships on the relationship maps (*N =* 26) that most support teacher well-being.

		+Closeness +Conflict (Quadrant I)	−Closeness, +Conflict (Quadrant II)	−Closeness, −Conflict (Quadrant III)	+Closeness −Conflict (Quadrant IV)
Male student	45	12	0	3	30
Female student	62	3	0	2	57
Total	107	15	0	5	87

Note: + = much; − = little; overall, 495 dyadic teacher–student relationships from 26 classes were located on the relationship map, including 244 (49.3%) female and 251 (50.7%) male students.

**Table 3:** Locations of dyadic teacher–student relationships on the relationship maps (*N* = 26) that most inhibit teacher well-being.

		+Closeness +Conflict (Quadrant I)	−Closeness, +Conflict (Quadrant II)	−Closeness, −Conflict (Quadrant III)	+Closeness −Conflict (Quadrant IV)
Male student	53 ^a^	18	17	13	2
Female student	21 ^b^	7	5	4	3
Total	74	25	22	17	5

Note: + = much; − = little; ^a^ 3 dyadic teacher–student relationships with male students were located at the intersection between Quadrants I and II; ^b^ 2 dyadic teacher–student relationships with female students were located at the intersection between Quadrants III and IV; 495 dyadic teacher–student relationships from 26 classes were located on the relationship map, including 244 (49.3%) female and 251 (50.7%) male students.

## Data Availability

The data presented in this study are available upon request from the corresponding author.

## Supplementary Materials

The following supporting information can be downloaded at https://www.mdpi.com/article/10.3390/ijerph20054053/s1. Table S1: Detailed coding scheme.
