# Supplementary material for: The Role of Dyadic Teacher–Student Relationships for Primary School Teachers’ Well-Being
Source: Int J Environ Res Public Health. 2023 Feb 24;20(5):4053. doi: 10.3390/ijerph20054053 (PMC10001482; doi:10.3390/ijerph20054053)
Supplement: Supplementary file 1 [file ijerph-20-04053-s001.zip › ijerph-2213611-supplementary.pdf]

Table S1. Detailed coding scheme.

Category	Subcategories	Short verbal examples of positive valence (+) and negative valence (-)
Relationship Schema Student		
Personality, intelligence, and special education needs	*Emotional stability*<br>Teacher describes the student with high (e.g., balanced, confident, calm) or low (e.g., moody, tense, worried, depressed, nervous, irritable) levels of emotional stability [76].	(+) "But he is so well-balanced" (P04, pos. 21)<br>(-) "Frustration tolerance equals to zero" (P20, pos 49)
	Extraversion<br>Teacher describes the student with high (e.g., sociable, assertive, energetic, active, enthusiastic) or low (e.g., quiet, not very talkative, shy) levels of extraversion [76].	(+) "Great charisma" (P24, pos. 12)<br>(-) "It's simple – there's just no output coming, no emotion from him, just really very little. Maybe I am a little disappointed in the sense of 'Hey, you could do so many cool things to talk about, also with the other kids, but you ONLY sit there and concentrate on yourself'. Maybe it's selfish of me too, but I just think that would be so nice" (P21, pos. 160)
	Agreeableness<br>Teacher describes the student with high (e.g., empathetic, warm, polite, selfless) or low (e.g., critical, lacking compassion, distrustful, harsh, unfriendly) levels of agreeableness [76].	(+) "Her friendly nature" (P03, pos. 90)<br>(-) "She has a sneaky character" (P06, pos. 48)
	Conscientiousness<br>Teacher describes the student with high (e.g., persistent, organized, determined) or low (e.g., impulsive, chaotic, careless) levels of conscientiousness [76].	(+) "Very dutiful child" (P01, pos. 86)<br>(-) No statements
	Openness<br>Teacher describes the student with high (e.g., broadly interested, imaginative, creative) or low (e.g., not very imaginative, not very creative) levels of openness [76].	(+) "I think from his side it has to do with the fact that he is very open" (P14, pos. 112)<br>(-) No statements

**Category**	**Subcategories**	**Short verbal examples of positive valence (+) and negative valence (-)**
	Sympathy<br>Teacher describes feelings of connectedness to the student or sympathy between the student and the teacher.	(+) "With this child I immediately had a good connection. The chemistry was right" (P05, pos. 70)<br>(-) "Maybe just personality. He is not so responsive to me" (P26, pos. 108)
	Intelligence<br>Teacher talks about the student's intelligence.	(+) "He is just clever. We can discuss or explain things at a slightly higher level than with other kids. It's just exciting to not only talk about the 4th grade material" (P21, pos. 122)<br>(-) No statements
	Special education needs<br>Teacher comments on special education needs of the student (e.g., confirmed or suspected diagnosis).	(+) "We are currently assessing whether he is autistic" (P14, pos. 146)<br>(-) No statements
Relational behavior	*Respect and discipline*<br>Teacher describes the extent to which the student shows a high degree (e.g., taking the teacher seriously, following the rules) or a low degree (e.g., being rude, not greeting, breaking the rules, testing the limits) of respect and discipline toward the teacher. If teacher speaks of "conflict" and does not specify the type of conflict, the passage is assigned to this category.	(+) "Almost too well-behaved" (P01, pos. 100)<br>(-) "What worries me is that it can trigger feelings in me so that I really almost explode. And how she can influence me with her behavior [...] That makes her really challenging. Sometimes I am standing here and she is doing something at her desk two steps away from me. Things like eating at school, or playing with her toys, even though we agreed that they are not allowed. She does that often in a row and really provokes me" (P19, pos. 105)
	Honesty<br>Teacher describes the extent to which the student shows a high degree or a low degree of honesty toward the teacher.	(+) "It is not that he does not do anything, but he came to me and was very honest. And that is exactly what I find really great" (P07, pos. 60)<br>(-) "She can lie straight to my face. And that hurts me" (P12, pos. 49)
	Clear communication of one's own needs<br>Teacher describes the extent to which the student clearly communicates her or his needs to the teacher.	(+) "Of course, that is very positive, that he explains me calmly, why he does not like to brush his teeth in class. That is not the same as someone moans, 'Ugh, that toothpaste!'" (P09, pos. 150)<br>(-) "If I cannot communicate, it is hard to build a relationship. With [name of student] it is difficult to communicate. There are ups and downs in our communication" (P03, pos. 114)

Category	Subcategories	Short verbal examples of positive valence (+) and negative valence (-)
	Trust<br>Teacher describes the extent to which the student does or does not trust the teacher.	(+) "I am happy when I know that she trusts me. Yes, that triggers a good feeling in me" (P19, pos. 83)<br>(-) "What I have also noticed, he has the feeling that I do not mean well when talking to him. He often accuses me of that, that I judge him badly, or of other things" (P23, pos. 129)
	Willingness to help<br>Teacher describes the extent to which the student behaves supportive, helpful, or loyal to the teacher.	(+) "When a few ladies complain that this topic is not interesting at all, she says directly: 'That is part of the school'. I can rely on her" (P25, pos. 38)<br>(-) "On the one hand understanding, and on the other hand just anger. Because I think she was fooling me during the parents' meeting, and at the same time I know that is not true, a child does not do that. It is really just about survival" (P11, pos. 134)
	Gratitude<br>Teacher describes the extent to which the student expresses gratitude to the teacher.	(+) "And when I do something, she is also grateful for it. On excursions, she is one of the few who says thank you" (P03, pos. 90)<br>(-) No statements
	Humor<br>Teacher describes the extent to which they experience humorous situations with the student. These can consist of laughing together, the student laughing at a teacher's jokes, or the student making funny comments.	(+) "She has a funny sense of humor, and we joke together" (P12, pos. 39)<br>(-) "We read a short text and then I asked: 'What does the text tell you?'. And then he said: 'The text does not tell me anything, it does not talk at all'. He does not realize in what situation it is funny and when it is not" (P25, pos. 54)
	Active relationship building with teacher<br>Teacher describes the extent to which the student engages in relationship building with the teacher (e.g., initiating conversations outside of the classroom).	(+) "I come into the classroom and he is already running toward me with something that he either wants to show me what he has done or that he wants to ask me (laughs)" (P14, pos. 108)<br>(-) "I think the distance only burdens me when I notice the student is not doing well. Then it burdens me. Because otherwise I find it completely okay and legitimate. I had to learn that there are children who do not like that. And yes, for example, with [name of student] I think it is quite all right like that" (P25, pos. 70)

Category	Subcategories	Short verbal examples of positive valence (+) and negative valence (-)
Motivational behavior	*Motivation*<br>Teacher describes the extent to which the student shows motivation (e.g., concentrates, has the materials with him or her, takes responsibility for his or her own learning).	(+) "I can rely on her, she is doing her best. And she also comes up with questions when she does not understand something. And that is a very good feeling for me. So I do not have to ask at home: 'Oh, why did she only do three tasks and they are all wrong?'" (P04, pos. 39)<br>(-) "Sometimes I get angry at the child because I think we have already discussed doing a reading comprehension once a week so many times. I asked, 'And, did you do it now?' and he answered 'No, I do not feel like it!'" (P25, pos. 22)
Socio-emotional behavior	*Interactions with classmates*<br>Teacher describes the extent to which the student demonstrates positive or negative social behaviors when interacting with peers.	(+) "That is what this class needs, maybe that is what makes me feel good, because she is so important, such a rock for the class. She holds the class together a bit, which is important" (P20, pos. 41)<br>(-) "One incident that shocked me was that he was fighting with a boy so much that he just lashed out. Then the grandmother of the other boy came [...] This boy threw the worst things at her like 'Your son deserves to die!' [...]. I did NOT know what to do anymore. And when you get into a situation like that, I realized that my relationship with him changed too. It's not his fault, it was a change. It was not against me, but in some ways, it was against me. It was extremely challenging, I had sleepless nights." (P20, pos. 49)
Performance behavior	*Performance*<br>Teacher describes the extent to which the student is performing well or poorly.	(+) "I cannot take credit for it, but I am proud of this girl. I can also praise her a lot and that's what we teachers extremely like to do" (P20, pos. 43)<br>(-) "But somehow I have the feeling that the child cannot mobilize its full resources. And that makes me a little nervous." (P20, pos. 43)

Category	Subcategories	Short verbal examples of positive valence (+) and negative valence (-)
Relationship Schema Teacher		
Personality	*Emotional stability*<br>Teacher describes their personality with high (e.g., balanced, confident, calm) or low (e.g., moody, tense, worried, depressed, nervous, irritable) levels of emotional stability [76].	(+) "Then I think maybe it has a little bit to do with my nature. Well, I am a very calm person and I am not someone who panics easily." (P24, pos. 66)<br>(-) No statements
	Extraversion<br>Teacher describes their personality with high (e.g., sociable, assertive, energetic, active, enthusiastic) or low (e.g., quiet, not very talkative, shy) levels of extraversion [76].	(+) No statements<br>(-) "I think I am more a distant person and do not allow too much closeness in the sense of privacy." (P08, pos. 18)
	Agreeableness<br>Teacher describes their personality with high (e.g., empathetic, warm, polite, selfless) or low (e.g., critical, lacking compassion, distrustful, harsh, unfriendly) levels of agreeableness [76].	(+) "I have a good sense for children" (P16, pos. 167)<br>(-) No statements
	Conscientiousness<br>Teacher describes their personality with high (e.g., persistent, organized, determined) or low (e.g., impulsive, chaotic, careless) levels of conscientiousness [76].	(+) No statements<br>(-) No statements
	Openness<br>Teacher describes their personality with high (e.g., broadly interested, imaginative, creative) or low (e.g., not very imaginative, not very creative) levels of openness [76].	(+) No statements<br>(-) No statements

**Category**	**Subcategories**	**Short verbal examples of positive valence (+) and negative valence (-)**
Relational behavior	*Honesty*<br>Teacher describes to what extent they behave honestly (e.g., admit own mistakes, show feelings openly).	(+) "I can also be open and apologize if I said something wrong: 'I am really sorry, I think I made a mistake here'" (P11, pos. 108)<br>(-) "It is still difficult to make it look real and not just a trick or an act" (P01, pos. 132)
	Understanding<br>Teacher describes the extent to which they meet the student with understanding (e.g., can understand the behavior).	(+) "Much of what the parents represent you then recognize in the child, including the problems that are passed on or lived through the child. And much can then be explained in the child. Sometimes it helps to find peace and say, 'Okay, I just need to show the child that there is something else'. And suddenly the child can develop. You know where the problem lies" (P11, pos. 200)<br>(-) "The desire for understanding, for a better understand of the child, is not fulfilled. I would like to understand, what does the child need? What does it want?" (P13, pos.93)
	Appreciation<br>Teacher describes the extent to which they are appreciative of the student (e.g., tolerant, listening, taking him or her seriously).	(+) "I think an important point is that I take him seriously" (P20, pos. 31)<br>(-) No statements
	Trust<br>Teacher describes the extent to which they trust the student.	(+) "I think we have that mutual trust" (P20, pos. 27)<br>(-) "I have not yet found a way to trust her" (P17, pos. 28)
	Humor<br>Teacher describes the extent to which they experience humorous situations with a student. These can consist of laughing together, a student laughing at a teacher's jokes, or a student making funny comments.	(+) "She comes up with funny remarks, and then I respond with other funny comments. We have developed our own humor together" (P12, pos. 39)<br>(-) No statements
	Reliability<br>Teacher describes the extent to which they are consistent or reliable with the student.	(+) "I would say she can rely on me, I think" (P01, pos. 98)<br>(-) No statements

Category	Subcategories	Short verbal examples of positive valence (+) and negative valence (-)
	Justice<br>Teacher describes the extent to which they behave fairly to the student.	(+) "Then I explained to the student: 'No, I am going to make a new impression of you. It is like I have a white sheet with nothing on it'. I think every kid deserves a chance" (P18, pos. 98)<br>(-) No statements
	Active relationship building with student<br>Teacher describes the extent to which they engage in relationship building with the student (e.g., initiating conversations outside of the classroom).	(+) "I just ask: 'How are you?' in the morning. Or: 'What did you eat?' Or: 'How is your hamster?' Or yes, often it is conversations like that in the morning when they come. I think that is where I can have a lot of influence" (P15, pos. 89)<br>(-) "Even though I am actually the teacher and should make the step toward them, I think I need a sign from the child like 'I need to be close to you or I want to chat a bit more'. And otherwise I tend to leave them alone because I find it artificial to act like 'best buddies' and I know 'No, it does not fit'. I wait for a sign that something is coming from him" (P08, pos. 52)
	Disciplinary interventions<br>Teacher describes the extent to which they intervene when the student causes disturbances.	(+) "Anger. I get really 'Ahh.' [...] I then explode. I really explode by saying 'It just does not work like that'. But then it is over. And then I am fine again" (P04, pos. 45)<br>(-) "I do not have to constantly intervene, everything just goes fine" (P18, pos. 22)
	Positive reinforcement<br>Teacher describes the extent to which they provide positive reinforcement (e.g., praise) to the student.	(+) "I cannot take credit for it, but I am proud of this girl. I can also praise her a lot and that is what we teachers extremely like to do" (P20, pos. 43)<br>(-) No statements
	(Professional) support<br>Teacher describes the extent to which they provide (professional) support to the student (e.g., formulating weekly goals, talking through mental health issues).	(+) "Challenge, self-reflection, frustration, but it is exactly this frustration that makes me reflect [...] It is on so many levels afterwards where you feel well-being, that you have [..] you have chosen the right path" (P13, pos. 75)<br>(-) "He is a totally interesting boy and I like him, really a lot. That is the dilemma. He is not well supported; he needs so much support that we cannot give him because he does not have the right for extra hours. We will probably get a class assistance because he is pretty much draining us with the attention he needs. […] That is where I do not feel comfortable anymore because I realize I am not doing justice to the other students, because I am

Category	Subcategories	Short verbal examples of positive valence (+) and negative valence (-)
		spending so much time on him. This is difficult for him and also for the other children" (P20, pos. 49)
Complementary professional strategies	*Parental involvement*<br>Teacher describes the extent to which they collaborate with the parents of the student.	(+) "With this child it was important that I had a good relationship with the mother first. I have the feeling that as soon as he knew 'Mommy says at home, it's ok at school, and the teacher does it well', then he was able to open up" (P23, pos. 101)<br>(-) "Lately I had a case where I really had to turn quite strongly away from the mother because I realized that her dislike of me was influencing my relationship with the student. I really had to take a pair of scissors in my hand, purely mentally, cut the umbilical cord and say: 'This is a person here, this has nothing to do with my student over there.' And that was hard. That was hardcore, honestly. I have never experienced that so strongly before, I was a bit scared" (P11, pos. 200)
	Collaboration with (special education) teachers and specialists<br>Teacher describes to what extent they collaborate with (special education) teachers and experts due to the student.	(+) "I was completely helpless. I needed help. So I got the school social worker on board" (P16, pos. 211)<br>(-) No statements
	Time to develop or improve the relationship<br>Teacher describes the extent to which spending time together with the student and its relation to the relationship quality.	(+) "The relationship also grows. At the end of the 6th grade, I have a better relationship with the children than at the beginning of the 5th grade" (P03, pos. 106)<br>(-) No statements
	Reset of the relationship<br>Teacher describes the extent to which they try to meet a student without reservations and give the relationship a new chance.	(+) "A principle of mine is 'Never start the day on yesterday's broken glass'. Every day is a new chance for every child. This is an important principle, and I have had good experiences with it. We can have conflicts, but the children also have to learn that they are over at one point and then it is good again" (P12, pos. 63)<br>(-) No statements
